# Berberine-loaded liquid crystalline nanoparticles inhibit non-small cell lung cancer proliferation and migration in vitro

**DOI:** 10.1007/s11356-022-19158-2

**Published:** 2022-02-16

**Authors:** Keshav R. Paudel, Meenu Mehta, Geena Hew Suet Yin, Lee Li Yen, Vamshikrishna Malyla, Vyoma K. Patel, Jithendra Panneerselvam, Thiagarajan Madheswaran, Ronan MacLoughlin, Niraj Kumar Jha, Piyush Kumar Gupta, Sachin Kumar Singh, Gaurav Gupta, Pradeep Kumar, Brian G. Oliver, Philip M. Hansbro, Dinesh Kumar Chellappan, Kamal Dua

**Affiliations:** 1 School of Life Sciences, University of Technology Sydney, Sydney, NSW 2007 Australia; 2 Centre for Inflammation, Centenary Institute, Sydney, NSW 2050 Australia; 3 Discipline of Pharmacy, Graduate School of Health, University of Technology Sydney, Sydney, NSW 2007 Australia; 4 School of Pharmacy, International Medical University, Bukit Jalil 57000, Kuala Lumpur, Malaysia; 5 Department of Pharmaceutical Technology, School of Pharmacy, International Medical University, Bukit Jalil 57000, Kuala Lumpur, Malaysia; 6 IDA Business Park, Dangan, H91 HE94 Galway Ireland; 7 School of Pharmacy & Biomolecular Sciences, Royal College of Surgeons in Ireland, Dublin, D02 YN77 Ireland; 8 School of Pharmacy & Pharmaceutical Sciences, Trinity College, Dublin, D02 PN40 Ireland; 9 Department of Biotechnology, School of Engineering & Technology (SET), Sharda University, Greater Noida, Uttar Pradesh, 201310 India; 10 Department of Life Sciences, School of Basic Sciences and Research (SBSR), Sharda University, Knowledge Park III, Greater Noida–201310, Uttar Pradesh, India; 11 School of Pharmaceutical Sciences, Lovely Professional University, Phagwara, Punjab 144411 India; 12 School of Pharmacy, Suresh Gyan Vihar University, Jagatpura 302017, Mahal Road, Jaipur, India; 13 Wits Advanced Drug Delivery Platform Research Unit, Department of Pharmacy and Pharmacology, School of Therapeutic Sciences, Faculty of Health Sciences, University of the Witwatersrand, Johannesburg, 2193 South Africa; 14 Woolcock Institute of Medical Research, University of Sydney, Sydney, NSW 2006 Australia; 15 Department of Life Sciences, School of Pharmacy, International Medical University, Bukit Jalil 57000, Kuala Lumpur, Malaysia; 16 Faculty of Health, Australian Research Centre in Complementary and Integrative Medicine, University of Technology Sydney, Ultimo, NSW 2007 Australia

**Keywords:** Berberine, Lung cancer, Liquid crystalline nanoparticles, Migration, Proliferation, Epithelial mesenchymal transition

## Abstract

Non-small cell lung cancer (NSCLC) is reported to have a high incidence rate and is one of the most prevalent types of cancer contributing towards 85% of all incidences of lung cancer. Berberine is an isoquinoline alkaloid which offers a broad range of therapeutical and pharmacological actions against cancer. However, extremely low water solubility and poor oral bioavailability have largely restricted its therapeutic applications. To overcome these limitations, we formulated berberine-loaded liquid crystalline nanoparticles (LCNs) and investigated their in vitro antiproliferative and antimigratory activity in human lung epithelial cancer cell line (A549). 3-(4,5-dimethylthiazol-2-yl)-2,5-diphenyl tetrazolium bromide (MTT), trypan blue staining, and colony forming assays were used to evaluate the anti-proliferative activity, while scratch wound healing assay and a modified Boyden chamber assay were carried out to determine the anti-migratory activity. We also investigated major proteins associated with lung cancer progression. The developed nanoparticles were found to have an average particle size of 181.3 nm with spherical shape, high entrapment efficiency (75.35%) and have shown sustained release behaviour. The most remarkable findings reported with berberine-loaded LCNs were significant suppression of proliferation, inhibition of colony formation, inhibition of invasion or migration via epithelial mesenchymal transition, and proliferation related proteins associated with cancer progression. Our findings suggest that anti-cancer compounds with the problem of poor solubility and bioavailability can be overcome by formulating them into nanotechnology-based delivery systems for better efficacy. Further in-depth investigations into anti-cancer mechanistic research will expand and strengthen the current findings of berberine-LCNs as a potential NSCLC treatment option.

## Introduction

Lung cancer is one of the most prevalent malignant tumours of the respiratory system, with significantly high rates of incidence and mortality (Khani et al. 2018). Lung cancer is responsible for about 30 out of every 100 cancer-related deaths (Siegel et al. 2021). In 2020 alone, there were more than 2 million new cases of lung cancer. In addition to this, there were also around 1.8 million deaths in the same year from lung cancer (Sung et al. 2021). About 85% of all cases of lung cancer are found to be non-small cell lung cancer (NSCLC) (Malyla et al. 2020). Smoking, pollution, urban development, and industrialization remain to be the leading causative factors for global incidences of lung cancer (Sharma et al. 2019). Surgical resection, radiation, chemotherapy, and immunotherapy are the primary treatments available for NSCLC (Bott et al. 2015, Bunn 2004, Klastersky &Awada 2012, Rolfo et al. 2014). Among these, chemotherapy has been one of the principal mainstays that constitutes the management of NSCLC. However, there have been several major concerns when it came to the safety and efficacy of the available chemotherapeutic agents. These concerns have provided a strong impetus, and the necessity to develop safer advanced novel therapeutic measures for the treatment and management of NSCLC.

Uncontrolled proliferation and migration are characteristic features of malignant lung cancer cells governed by a variety of signalling pathways, which culminate in the nucleus to regulate vital cellular transcription processes. Genes that enhance the proliferation or migration of cells are generally upregulated in lung cancer cells when compared to normal cells (Millar et al. 2017). Epidermal growth factor receptor (EGFR) and AXL belong to the group of tyrosine kinases, which are overexpressed in lung cancer and their activation promotes tumour development, invasion, and metastasis (Sigismund et al. 2018). Upregulation of EGFR and AXL activates subsequent pro-oncogenic signalling pathways such as the Ras-Raf-mitogen-activated protein kinase (MEK)-mitogen-activated protein kinase (MAPK) and the phosphoinositide 3-kinase (PI3K)-Akt/protein kinase B-mammalian target of rapamycin (mTOR). AXL promotes cell survival by modulating nuclear factor kappa-light-chain-enhancer of activated B cells (NFkB) nuclear translocation, enhancing anti-apoptotic marker expression [survivin, B-cell lymphoma-2 (BCL-2), and BCL-extra large (BCL-XL)], and decreasing the activity of pro-apoptotic proteins (BCL2 associated agonist of cell death and caspase-3) (Hasanbasic et al. 2004). These pathways then activate a number of physiological mechanisms that aid cancer cell growth, such as initiation, chronic maintenance, and progression through the cell cycle (Laskin &Sandler 2004).

Platelet-derived growth factor-AA (PDGF-AA) is well-reported to be the most potent tumour angiogenesis mediator (Noskovičová et al. 2015). PDGF-AA acts as an autocrine regulator of vascular endothelial growth factor (VEGF) expression, facilitating the transformation of precancerous lesions into metastatic cancer (Shikada et al. 2005). It has been proven that galectin-3 increases VEGF-mediated angiogenesis (Markowska et al. 2010). Progranulin, another glycoprotein secreted by endothelial cells that controls cell proliferation, migration, and survival (He and Bateman 1999). Progranulin overexpression in endothelial cells affects normal angiogenesis in vivo (Toh et al. 2013).

Epithelial-mesenchymal transition (EMT) is the early stage of tumour metastasis in which the epithelial cells lose their cellular adhesion and polarity while gaining migration and invasive properties (Thiery et al. 2009). E-cadherin (an epithelial cell marker) is drastically downregulated during EMT, whereas N-cadherin and Vimentin (mesenchymal cell markers) are increased (Kidd et al. 2014). Zinc finger protein SNAI1 (SNAIL) has been identified as a transcriptional factor that promotes EMT by suppressing the adhesion protein, i.e. E-cadherin (Wang et al. 2013), whereas p27 promotes epithelial–mesenchymal transition by upregulating signal transducer and activator of transcription-3-mediated Twist1 (Zhao et al. 2015). Cathepsin S, a lysosomal cysteine protease, can breakdown extracellular matrix and thereby accelerate tumour migration. (McDowell et al. 2020).

**Fig. 1:**
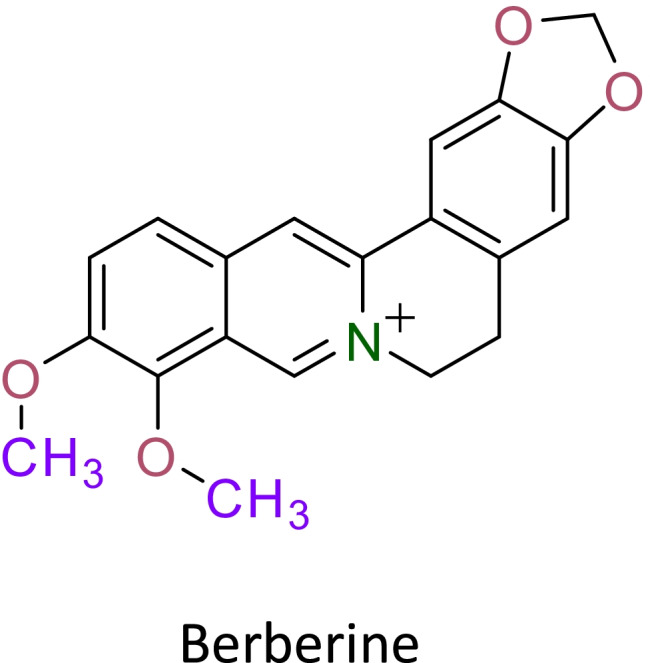
The chemical structure of berberine

Berberine is an iso-quinoline alkaloid (Fig. 1) obtained from the plant families such as Ranunculaceae and Papaveraceae (Bhardwaj &Kaushik 2012). A significant number of studies in the past have shown its antitumor potential against various cell lines and xenograft models (Chang et al. 2020; Mittal et al. 2014; Singh et al. 2011; Wang et al. 2016). Synthetic derivate of berberine such as dimethylberberine inhibits reactive oxygen/nitrogen species, mitochondrial dysfunctions, and inflammation mediators such as NFkB, tumour necrosis factor-α, interleukin (IL)-6, and IL-8 (Gupta et al. 2021). While berberine has several benefits in the treatment of various diseases, its applicability for any therapeutic agent is restricted by several limitations, i.e. poor oral bioavailability, low gastrointestinal absorption, and a high degree of elimination (Liu et al. 2016; Spinozzi et al. 2014). In order to achieve the intended therapeutic outcome, efforts must be made to improve its solubility, oral bioavailability, and maintain a consistent plasma concentration.

Nano-based drug carrier systems are now being employed as one of the most effective techniques in cancer chemotherapy (Ng et al. 2020). Nanoformulation of natural product-derived compound such as curcumin (Kabir et al. 2021), berberine (Mehta et al. 2021a), boswellic acid (Solanki et al. 2020) has shown promising anticancer activity against various cancers including lung cancer (Bhattacharya et al. 2021). Advances in nanotechnology-based medical appliances, diagnostic tools, therapeutic approach, and novel vaccine production has created hope to discover new management strategy of various cancers (El-Sayed &Kamel 2020). Among nanoformulations, liquid crystalline nanoparticles (LCNs) have gained a lot of interest in the pharmaceutical sector because of their extraordinary potential to improve medication bioavailability and enhanced stability of the active drug component. In addition, these nanostructures have the inherent ability to alter the release of drug when administered through various routes (Paudel et al. 2021). Monoolein (MO) is a type of lipid used in the formulation of LCNs. The distinctive construct of MO enables LCNs to incorporate a wide range of drugs with varying solubilities (Dawoud &Nasr 2016). The principal goals of our study revolved around formulating berberine-loaded MO-based LCNs (MO-LCNs) and subsequently study the anti-cancer potential of the prepared formulation in a human lung epithelial carcinoma (A549) cell-line model. We hypothesized that berberine-LCNs formulation would have improved physiochemical characterization that could possibly favours cellular uptake and therefore the formulation would show potent anti-cancer activity as low dose compared to the high dose of free berberine for similar activity in published literature.

## Materials and methods

Berberine hydrochloride, monoolein, and poloxamer 407 (P407) were procured from Sigma Chemicals Co, Germany. Spectra/Por dialysis membrane bags consisting of cellulose membrane dialysis tubing (14,000 g/mol molecular-weight-cut-off were procured from Sigma-Aldrich, USA. Additional chemicals, solvents, and reagents utilized were of analytical or spectroscopic grade that were not purified further.

### Formulation development using QbD approach

#### Optimisation of formulation parameters: three-level factorial design

Critical quality attributes, critical material attributes, and critical process parameters were identified through pre-existing literature to construct a design space where the relationships among the independent variables (concentration of MO and sonication time) were analysed using three-level factorial design with response surface methodology to comprehend the effects and interactions of particle size and entrapment efficiency of LCNs produced. Design-Expert software version 12 was used to evaluate the data and develop full model equations and contour plots based on the varied levels of independent variables. A total of nine formulations (BM1-BM9) were prepared as per three level factorial design (Table 1).

**Table 1:** Compositions of formulations of BBR-MO-LCNs and their characterization parameters

Formulation	Conc. of MO (% w/w)	Conc.of P407 (% w/w) *	Berberine hydrochloride (% w/w)	Water	Sonication amplitude (%)	PS (nm)	EE (%)
B-M1	1	10	0.01	Up to 5 mL	20	147.1	75.31
B-M2	2	10	0.01	Up to 5 mL	20	148.2	73.72
B-M3	4	10	0.01	Up to 5 mL	20	170.9	73.72
B-M4	1	10	0.01	Up to 5 mL	40	131.2	71.49
B-M5	2	10	0.01	Up to 5 mL	40	139.7	74.11
B-M6	4	10	0.01	Up to 5 mL	40	149.3	72.12
B-M7	1	10	0.01	Up to 5 mL	80	143.9	73.16
B-M8	2	10	0.01	Up to 5 mL	80	139.2	73.65
B-M9	4	10	0.01	Up to 5 mL	80	191.4	74.53

*PS* particle size, *EE* entrapment efficiency

#### Preparation of berberine-LCNs

**Fig. 2:**
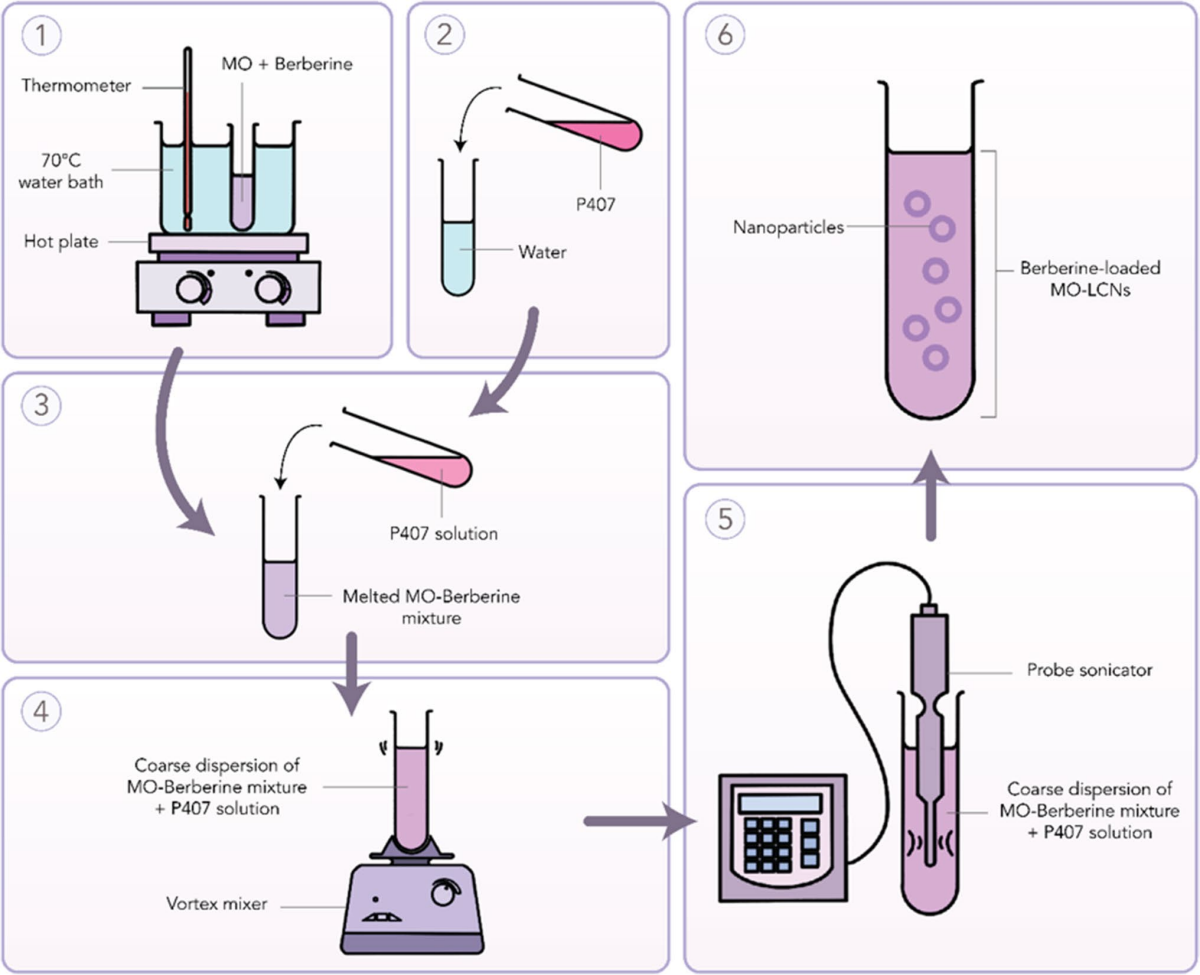
Pictorial description for the preparation of berberine-LCNs

We employed the ultrasonication technique to formulate the berberine-loaded nanoparticles. Firstly, we weighed 200 mg of MO in a glass vial. In another glass vial, we dissolved 20 mg of P407 in 4.8 mL of water. These vials were then heated at 70 °C in a water bath set-up. Secondly, 5 mg of berberine was weighed and was then added into the vial that contained the melted MO. The mixture was vortexed until complete dissolution. The surfactant solution (containing P407) was then mixed with the MO-drug mixture. This resulted in the formation of a coarse dispersion. Finally, the resultant coarse dispersion was treated to size reduction employing a probe sonicator (Labsonic® P, Sartorius, Germany). The amplitude was maintained at 80 along with 5-s on and 5-s off-cycles for a period of 5 min. As a result, 5 mL of 1 mg/mL berberine-loaded MO-LCNs were prepared (Fig. 2). The lipid content of the prepared formulation was 40 mg/mL. The lipid:surfactant ratio of the formulation was 1:10 wt/wt. A similar protocol was used to make blank LCNs but without the inclusion of berberine. The inherent molecular interactions existing within the nanosystems were extracted and analysed using atomistic simulations in vacuum through a conjugate gradient within an MM + force field as described elsewhere (ChemLite 30 Molecular Modelling Software) (Fig. 1) (Chan et al. 2020; Paudel et al. 2021).

### Physicochemical characterization

#### Particle size, polydispersity index (PDI), and zeta potential

The particle size, PDI, and zeta potential of the berberine LCNs were measured using a Zetasizer Nano ZS (Malvern Instruments, Malvern, UK). The samples were diluted (20-fold) with distilled water before analysis. All data were measured in triplicate at 25 °C (Chan et al. 2020; Jin-Ying et al. 2020; Ng et al. 2018).

#### Entrapment efficiency (EE)

The EE of the LCNs dispersion was measured by ultracentrifugation method. A total volume of 500 µL berberine LCNs dispersion was centrifuged via a high-speed centrifuge (Eppendorf Centrifuge 5810R, Germany) at 4000 rpm at 25 °C for a 15-min duration. The filtrate and lysed dispersion were evaluated using UV–Vis spectrophotometer (UV-1800, Shimadzu AS, Japan) at *λ* = 257 nm to determine the free drug (*D*_free_) and total drug (*D*_total_) concentration. In three replicates, the EE was calculated using the equation below:

$$\%EE =\frac{{D}_{\mathrm{total}}-{D}_{\mathrm{free}}}{{D}_{\mathrm{total}}}\times 100$$

where, the entrapment efficiency is represented by EE; the total amount of drug in the prepared formulation is represented by *D*_total_, and the total free drug in the prepared formulation is represented by *D*_free_ (Chan et al. 2020; Jin-Ying et al. 2020; Ng et al. 2018).

#### Morphology

One drop of diluted (50-fold dilution) berberine LCNs was loaded onto a carbon-coated copper grid. This was allowed to air-dry before the inspection. The inspection was carried out using a transmission electron microscope (Fischione Instrument Inc, USA).

#### In vitro release study

We employed the dialysis bag method to study the in vitro release of the drug from the formulation. Spectra/Por dialysis membrane bags were submerged in the buffer overnight before use. The bags loaded with 2 mL samples were clamped at both ends prior to immersion in 30 ml release media (phosphate buffer, pH 7.4 ± 0.2) in Falcon® conical tubes (Corning, NY, USA). The falcon tubes were immersed in a water bath (SW22 Julabo) maintained at 37 °C. These tubes were horizontally shaken at 50 strokes/min. At regular time intervals of 0.5, 1, 2, 4, 6, 8, 10, 12, 24, 48, and 72 h, an accurately measured volume of 1 mL of the sample solution was withdrawn from the tube for analysis at time points. The sample withdrawn was reimbursed with the same volume of dissolution media. Berberine release was quantitatively determined by UV–Vis method, at 257 nm (Jin-Ying et al. 2020, Paudel et al. 2021).

### Cell culture and reagents

Cell culture studies were performed using the A549 (human lung epithelial carcinoma) cell line (ATCC, USA) which was provided as a kind gift by Prof. Alaina Ammit, at the Woolcock Institute of Medical Research, Sydney, Australia. A humified 37 °C incubator was used to grow the cells. The cells were cultured in 5% CO_2_ incubator using Dulbecco’s Modified Eagle’s Medium (DMEM) supplemented with 10% fetal bovine serum, 1% penicillin, and streptomycin. MTT (3-[4,5-dimethylthiazol-2-yl]-2,5-diphenyl tetrazolium bromide), crystal violet, dimethyl sulphoxide (DMSO), haematoxylin and eosin (H&E) staining solution were procured from Sigma-Aldrich, St. Louis, MO, USA. The human XL oncology array kits (R&D systems, USA) were purchased from in vitro technologies pvt ltd, Australia. Additional chemical reagents and consumables were obtained from Sigma-Aldrich unless specified.

### Proliferation assay

#### MTT assay (cell viability assay)

The MTT assay was used to assess the anti-proliferative activity of berberine LCNs, as previously described by Paudel et al. (Lee et al. 2015). The A549 cells were seeded in a 96-well plate. After attachment, these cells were subjected to the treatment with berberine LCNs. Various concentrations namely, 0.5, 1, 2.5, and 5 μM were treated to A549 cells for 24 h. Subsequently, 10 µl of MTT solution (5 mg/ml in PBS) was added to each well and cells were incubated for a period of 4 h inside the incubator. After the supernatant was removed, we added 100 μL DMSO to solubilize the purple colour formazan. The absorbance for this product was measured at 540 nm using BMG Labtech microplate reader (POLARstar Omega, Australia). The control group’s proliferation rate was set to 100%, and the percentage (%) proliferation of berberine LCNs-treated cells was calculated.

#### Trypan blue staining

The trypan blue assay was performed using 48-well plates. A549 cells were seeded at a density of 20,000 cells per well. The cells were treated with berberine-LCNs at concentrations of 0.5, 1, 2.5, and 5 μM for 24 h. The cells were then detached with trypsin followed by centrifugation for 4 min at 1200 rpm to collect cell pellets. Subsequently, a 0.4% trypan blue solution was mixed with cells at 1:1 ratio (10 μl each). Number of live cells along with the total cell count were counted under a light microscope at a magnification of 10X (Ahmad et al. 2006).

### Wound healing assay

This assay was performed in 6-well plates. A549 cells were seeded at a concentration of 3 × 10^5^/well. The assay was performed based on the same method reported earlier (Jun et al. 2016). The cells were further grown until they formed a monolayer. A scratch wound was created with a tip of a 200 μl sterile pipette. This step was followed by washing the layer with PBS to remove the detached cells. Images were captured at time 0, and subsequently berberine-LCNs was treated for 24 h at various concentrations of 0.5, 1, 2.5, and 5 μM. The migrating cells in the wound area were captured with the help of a light microscope using a magnification of 10X. The distance between the monolayer’s two edges was measured and the wound closure percentage was eventually calculated.

### Boyden’s chamber assay

The migration properties in the A549 cells were evaluated using a modified Boyden’s chamber assay. In this assay, Transwell permeable supports (6.5-mm insert, 8-µM pore size polycarbonate membrane) were used. The outer surface of the transwell membrane was coated with 2.5% gelatin in 1 M acetic acid and left to dry for an hour. In the upper compartment, A549 cells were seeded at a density of 1 × 10^4^ cells/ml and kept in a well of 12 well plates with DMEM (600 μl). After 24 h, the cells were treated with berberine-LCNs and allowed to migrate for another 24 h. Cotton wipes were used to cleanse the non-migrated cells that remained on the top surface of the membrane. H&E were used to stain the cells that migrated to the membrane’s bottom surface. These cells were fixed in 10% formalin prior to the staining. The membranes were then put on microscope slides. The cells that escape across the membrane pores were counted at 20X magnification in five random fields. The average cells per field of view were then calculated (Paudel et al. 2016).

### Colony formation assay

Six-well plates were used for this assay. A549 cells were plated at a density of 500 cells per well. After attachment, the cells were treated with berberine-LCNs and allowed to colonize for 2 weeks while changing the media every 48–72 h. After colonization, the wells were rinsed with PBS and then fixed for 20 min in 3.7% formaldehyde. After washing the cells with PBS solution, cells were stained with 0.4% crystal violet and washed again multiple times with PBS. Six-well plates were inverted and images of six-well plates were taken (Gour et al. 2019).

### Human oncology protein array

A549 cells (3 × 10^5^ cells/well) were seeded in 6-well plates. The cells were treated with or without different concentrations of berberine-LCNs for a 24 h period. The total cellular protein was extracted using radioimmunoprecipitation assay lysis buffer containing protease and phosphatase inhibitor cocktail which was further quantified by a bicinchoninic acid assay kit. A total of 350 μg of protein was used for both control and treatment groups to perform an oncology array (R&D Systems, Minneapolis, MN) as per the manufacture’s protocol (Argentiero et al. 2019).

### Statistical analysis

All observations were documented as mean ± SEM. All experiments were performed in triplicate. A 2-tailed Student’s *t*-test was performed to calculate the statistical significance of data between 2 groups. Furthermore, one-way ANOVA followed by Dunnett’s multiple comparison test was employed where data belonging to more than 2 groups was analysed. We used a Graph Pad Prism software (version 8.2.1) to perform the statistical analyses. Significance in the statistical data was accepted at *P* < 0.05.

## Results

### Preparation and optimization of berberine-LCNs

**Fig. 3:**
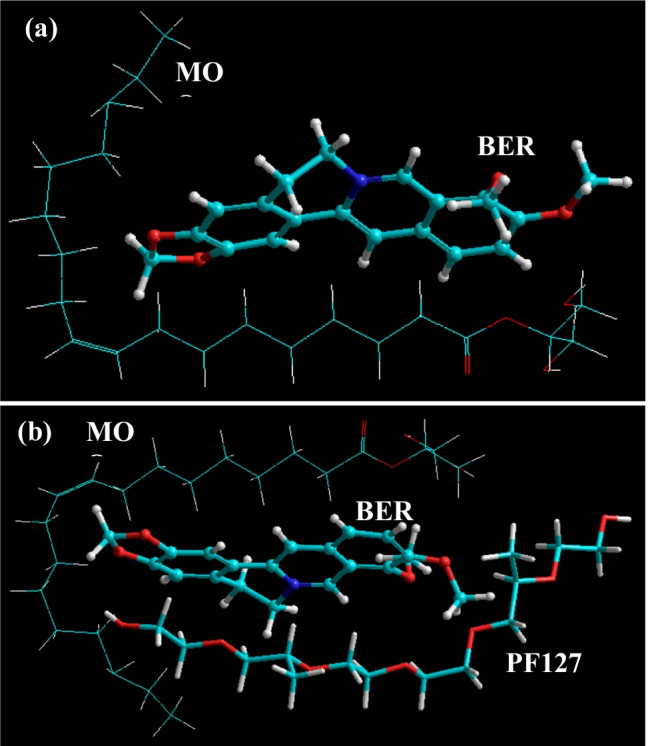
Molecular mapping and geometrical positioning of berberine with **a** monoolein, and **b** monoolein and PF127, after molecular mechanics simulations in vacuum

The berberine-LCNs were produced as a yellowish, cloudy dispersion mixture, whereas the blank MO-LCNs were presented as a white, cloudy dispersion. The molecular simulation of the bimolecular (BER-MO) and trimolecular (BER-MO-PF127) systems revealed similar energy stabilization patterns characterized by initial destabilization of the drug-excipient complexes along with stabilization of attractive van der Waals interactions reaching negative energy values of − 7.522 and − 25.343 kcal/mol, respectively (Table 2). A close look at the geometrical orientation confirms the preferable positioning of the drug within the lipid molecule (Fig. 3a), which further got enclosed into the MOO-PF127 system under formulation conditions (Fig. 3b).

**Table 2:** Molecular attributes and corresponding energy values for various drug-excipients in silico complexes

Molecular attributes	BER	MO	BER-MO	PF127	B-M-PF
Total energy	30.37	3.29	90.364	29.249	100.132
Bond energy	1.22	0.213	1.58	0.371	1.996
Angle energy	11.764	1.796	80.39	1.771	81.743
Dihedral angle energy	12.007	1.501	15.916	25.169	41.735
van der Waals energy	5.377	− 0.221	− 7.522^#^	1.937	− 25.343^#^

All energy values are in kcal/mol. ^#^High energy stabilizing component

**Fig. 4:**
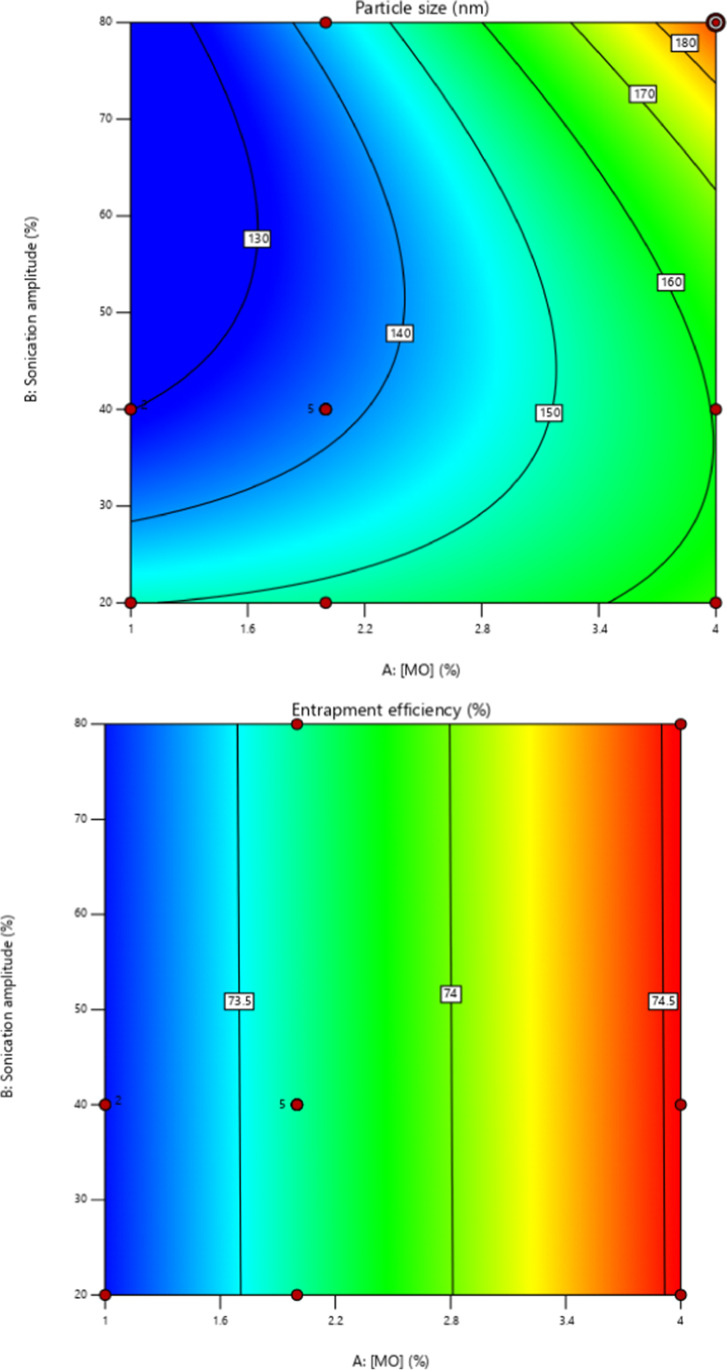
**a** Contour plots of PS for berberine-loaded MO-LCNs. **b** Contour plots of EE for berberine-loaded MO-LCNs

The parameters used for preparation of BBR loaded MO-LCNs are listed in Table 1. The overall results showed that the PS ranged from 131.2 to 191.4 nm and the EE ranged from 75.31% to 71 respectively. 3D response surface (Fig. 4) and polynomial equation generated using Design Expert® Software 12. The finalized reduced quadratic and linear equations with respect to the variables for PS (Y1) and EE (Y2) are shown in Eqs. (1) and (2) respectively. ANOVA results of the models adopted for both variables were appropriate in stipulating the significance of its effects whereby their *p*-values were lesser than 0.05.

1 $$Y1=185.70188-5.69437\mathrm{ A}-2.11907\mathrm{ B}+0.270458\mathrm{ AB}+1.00610 A2+0.014256 B2$$

2 $$Y2=72.72559-0.452422\mathrm{ A}+0.000124\mathrm{ B}$$

Both factors A and B of multiple regression Eq. 2 showed negative effects on the PS of BBR-MO-LCNs where an increase in A, reduces the PS. Factors AB, A^2^ and B^2^ showed positive effects where increase of these factors, increased the PS. The effects of factors A in multiple regression Eq. 3, a linear model, showed negative effects whereas factor B showed positive effects in terms of EE. The independent variable A was shown to be significant whereas variable B was insignificant for prediction of EE.

Out of nine formulations first formulation comprising of 4% MO concentration and 80% sonication amplitude was selected as the optimized formulation due to its higher desirability. The optimized formulation was developed using ultrasonication method.

### Physicochemical characterization of optimized formulation

Table 3 summarizes the characterisation parameters of both blank and berberine-LCNs. The average particle size of blank and berberine-LCNs was 178.5 and 181.3 nm, respectively, as per dynamic light scattering characterization. The PDI for both formulations was less than 0.1 and the zeta potential was less than − 5 mV. Furthermore, encapsulation efficiency results showed that about 75% of berberine was encapsulated in MO-LCNs.

**Table 3:** Characterisation parameters of blank and berberine-LCNs

	**Blank MO-LCNs**	**Berberine-LCNs**
Z-average (nm)	178.5 ± 1.8	181.3 ± 0.7
Polydispersity index (pdi)	0.129 ± 0.010	0.075 ± 0.010
Zeta potential (mV)	− 9.83 ± 0.371	− 5.19 ± 0.214
Encapsulation efficiency (%)	-	75.35 ± 0.005

**Fig. 5:**
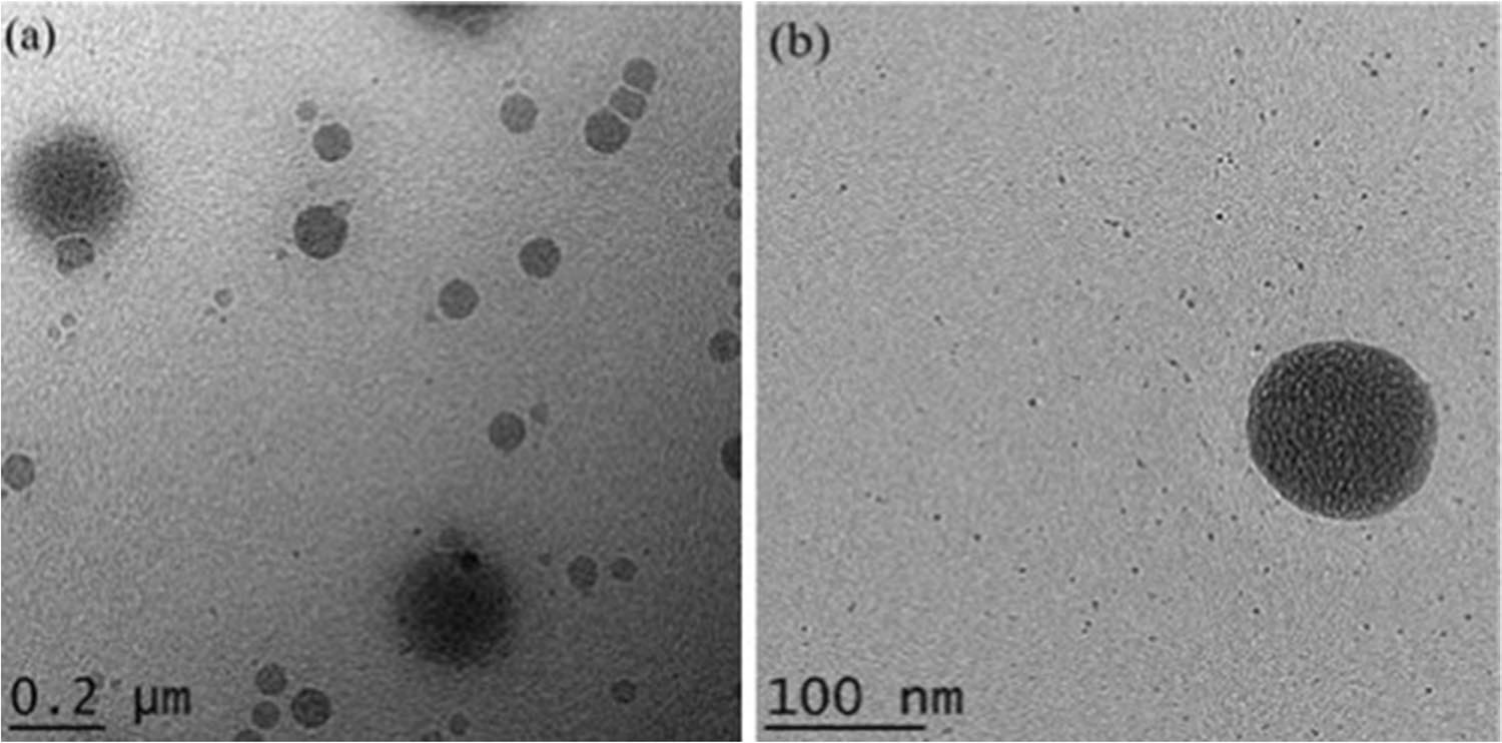
**a** TEM image of berberine-loaded MO-LCNs with a scale bar of 200 nm; **b** TEM image of berberine-loaded MO-LCNs with a scale bar of 100 nm

**Fig. 6:**
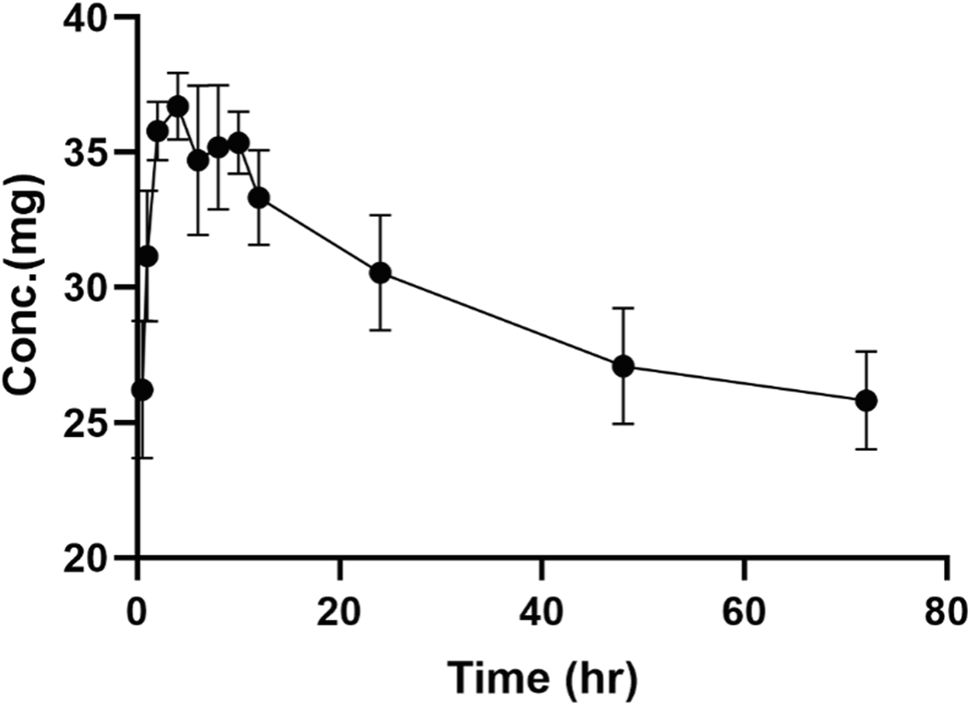
In vitro release profile of berberine LCNs

The transmission electron microscopy (TEM) visualization (Fig. 5a and b) revealed that berberine-LCNs were small, smooth surfaced, and spherical in shape and were monodispersed in uniform size. The in vitro release profile of berberine-LCNs (Fig. 6) showed a more rapid release as compared to the standard during the initial phase. The rapid release was followed by a plateau phase but the cumulative berberine release for both the standard and berberine-LCNs exceeded 100%. The plotted graph suggested a first-order drug release kinetics and a biphasic release pattern.

### Anti-proliferative activity of berberine-LCNs in A549 cells

**Fig. 7:**
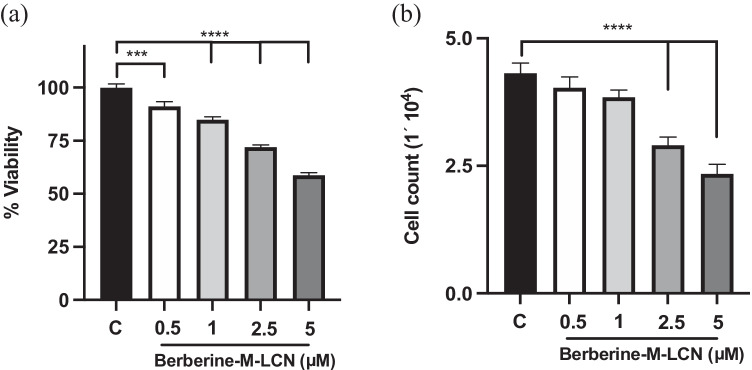
Anti-proliferative activity of berberine-LCNs in A549 cells. **a** MTT cell viability assay. **b** Cell count after trypan blue staining. ***p < 0.001, ****p < 0.0001 vs control (without berberine-LCNs treatment). Values are expressed as mean ± SEM, *n* = 3 independent experiments. Analysis was done by one-way ANOVA followed by Dunnett’s multiple comparison test

The effect of berberine-LCNs on the A549 cell proliferation is presented in Fig. 7a and b. Berberine-LCNs, at doses of 0.5, 1, 2.5, and 5 μM, reduced the rates of proliferation to 8.8%, 15.19%, 28.10%, and 41.29%, respectively of the control (without berberine-LCNs treatment) in the MTT assay. The IC_50_ value was 10.1 μM. Moreover, the rates of cell proliferation were reduced to 6.5%, 10.86%, 32.6%, and 45.65% respectively of the control (without berberine-LCNs treatment) in the Trypan blue staining cell count.

### Anti-migratory activity of berberine-LCNs in A549 cells

**Fig. 8:**
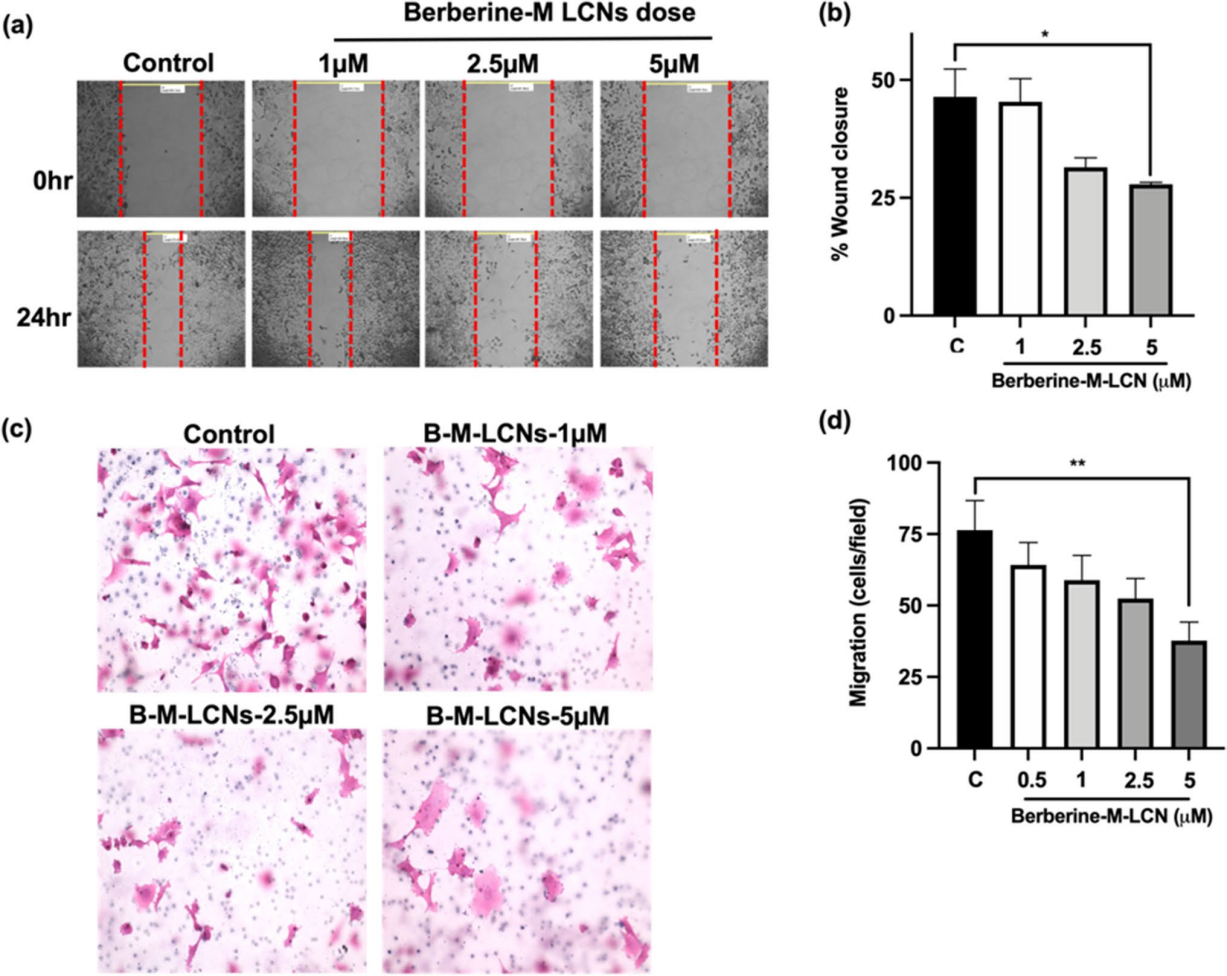
Anti-migratory activity of berberine-LCNs in A549 cells. **a** Wound was created by scratching with a sterile tip on a confluent A549 in six-well plate and treated with or without various doses of berberine-LCNs for 24 h. Photographs were taken on 10 × magnification. **b** The distance between two edges of wound was measured for 0 and 24 h to calculate the percentage wound closure. **c** A549 cells were seeded in a transwell chamber and treated with or without various doses of berberine-LCNs. Cells were allow to migrate through the membrane for 24 h. Migrated cells were stained with haematoxylin and eosin and photographed under a microscope. **d** The cells on the outer layer of the membrane after migration were counted in 5 random positions under a high-power field. Values are expressed as mean ± SEM (*n* = 3 independent experiments); *p < 0.05, **p < 0.01 vs control (without berberine-LCNs treatment); magnification 20 × . Analysis was performed by one-way ANOVA followed by Dunnett’s multiple comparison test

Wound healing assay and Transwell Boyden chamber assays were used to evaluate the effects of berberine-LCNs on A549 cell migration. Our findings are presented in Fig. 7a–d. There was a dose-dependent suppression by berberine-LCNs in the A549 cell migration as observed in the wound healing for 24 h (Fig. 8a). The magnitude of the inhibition with 1, 2.5, and 5 μM of berberine-LCNs was approximately 2%, 32%, and 40% respectively. These observations were also compared with the control (Fig. 8b). In addition to the above, berberine-LCNs also significantly inhibited the migration of A549 cells as observed in the Transwell chamber assay (Fig. 8c–d). Berberine-LCNs, at a dose of 1, 2.5, and 5 μM, inhibited A549 migration by 12.19%, 23.9%, 28.78%, and 55.6%, respectively (Fig. 8d).

### Anti-colony formation activity of berberine-LCNs in A549 cells

**Fig. 9:**
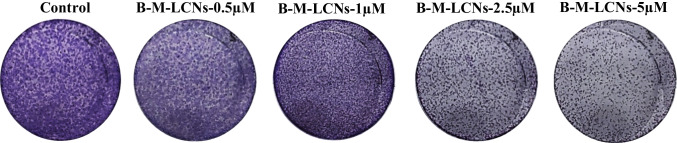
Colony formation activity of berberine-LCNs in A549 cells. A549 cells were seeded in a six-well plate and treated with or without various doses of berberine-LCNs for 24 h. Cells were stained with crystal violet staining solution, after which the six-well plate was inverted to capture the image of an individual well

The crystal violet staining method was employed to determine the anti-colony forming activity of berberine-LCNs in A549 cells. As shown in Fig. 9, there was a dose dependent inhibition exhibited by berberine-LCNs over colony formation in A549 cells.

### Inhibition of EMT related protein expression by berberine-LCNs

**Fig. 10:**
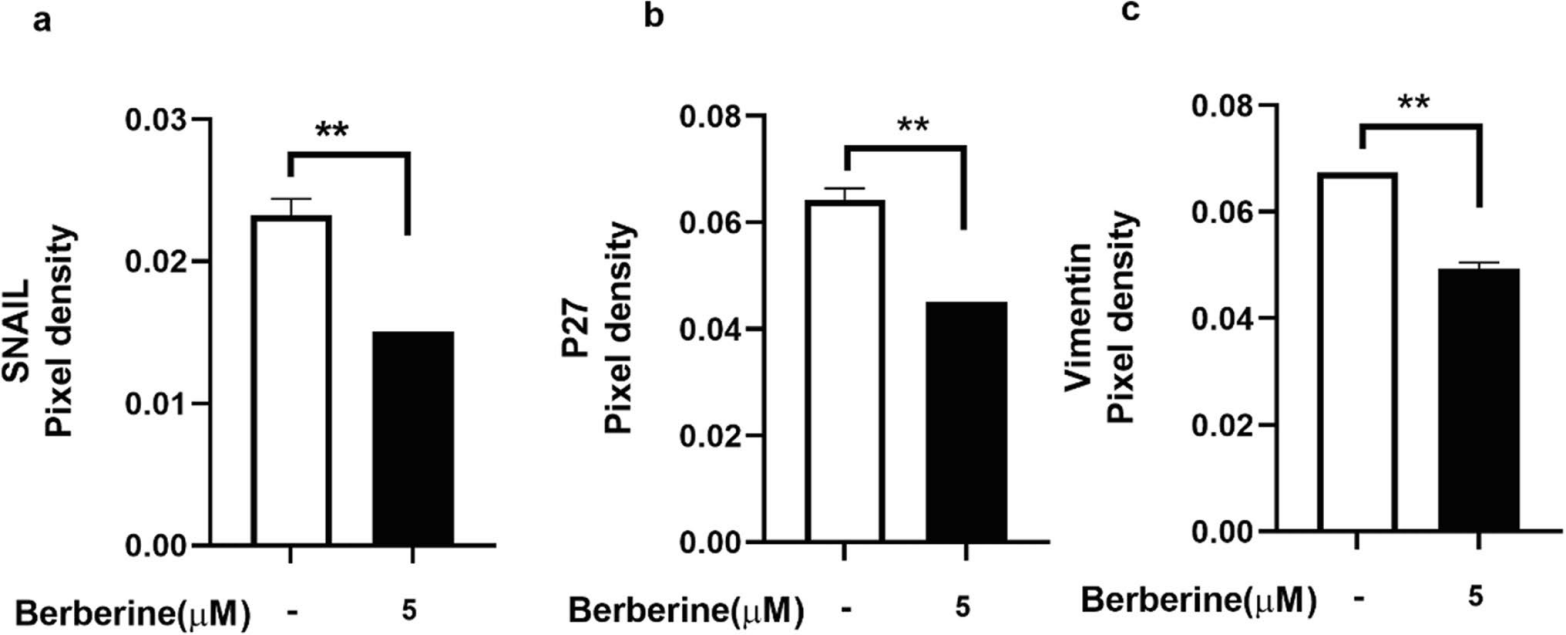
Inhibition of expression of EMT related proteins **a** SNAIL, **b** P27, and **c** Vimentin upon treatment with berberine-LCNs on A549 cells. Values are expressed as mean ± SEM (*n* = 2); **P < 0.01 vs control (without berberine-LCNs treatment). Analysis was performed by a 2-tailed Student’s *t*-test

Cancer cell metastasis is facilitated by EMT-related proteins (Xiao &He 2010). The effect of berberine-LCNs on SNAIL, P27, and Vimentin protein expression in A549 cells is shown in Fig. 10a–c. It was observed that the expression of all three EMT-related proteins, SNAIL, P27, and Vimentin was significantly inhibited by berberine-LCNs (Fig. 10a–c).

### Inhibition of migration and proliferation related protein expression by berberine-LCNs

**Fig. 11:**
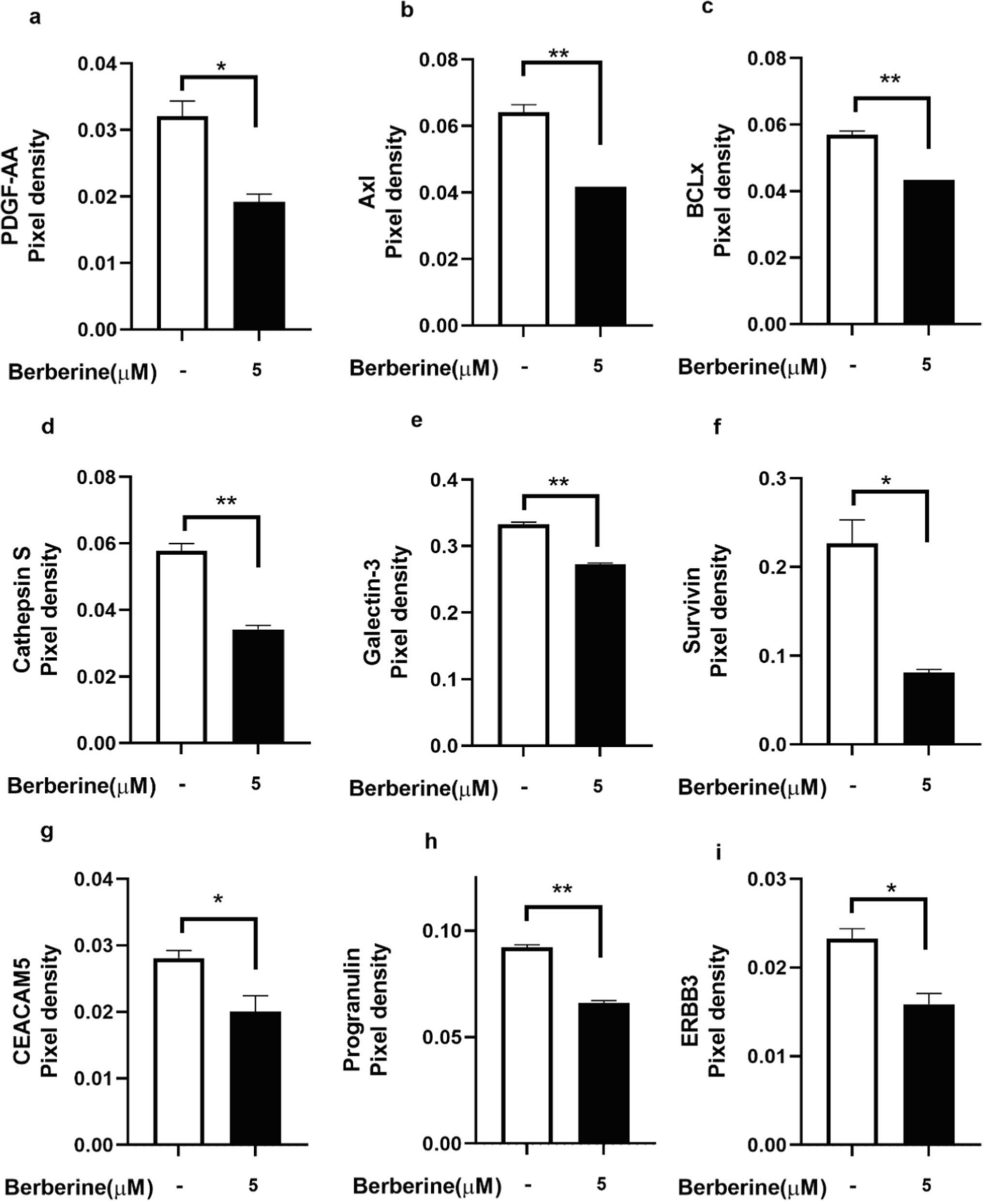
Inhibition of expression of protein **a** PDGF-AA, **b** Axl, **c** BCLx, **d** Cathepsin S, **e** Galectin-3, **f** Survivin, **g** CEACAM5, **h** Progranulin, and **i** ERBB3 upon treatment with berberine-LCNs on A549 cells. Values are expressed as mean ± SEM (*n* = 2); *p < 0.05, **p < 0.01 vs control (without berberine-LCNs treatment). Analysis was performed by a 2-tailed Student’s *t*-test

Several proteins are involved cell signalling pathway leading to lung cancer proliferation and migration (Yang et al. 2017a). The effect of berberine-LCNs on *PDGF-AA*, *Axl*, *BCLx*, *Cathepsin S*, *Galectin-3*, *Survivin*, *CEACAM5*, *Progranulin*, *and ERBB3* protein expression in A549 cells is shown in Fig. 11a–i. It was observed that the expression of all proliferation and migration-related proteins was significantly inhibited by berberine-LCNs.

## Discussion

In our in vitro study, we have revealed the potent beneficial activity of berberine-LCNs against human lung cancer cell line (A549). The most notable activity observed with berberine-LCNs were significant inhibition of proliferation, colony formation, invasion/migration, and protein expression associated with these cancer progression events.

Researchers have widely explored various herbal plant extracts and their active chemical constituents for their potent anticancer activities, including lung cancer (Hardwick et al. 2021; Panth et al. 2017; Solanki et al. 2020). In this study, we have investigated a widely known and commercially available anti-inflammatory compound berberine to investigate if it possesses anticancer potential against A549 cells. Berberine has very low dissolution rate and oral bioavailability owing to the fact that it is very slightly soluble in water, hence limiting its clinical application (Singh et al. 2020). Due to poor bioavailability, the oral dosage of berberine prescribed for daily treatment of certain disease conditions ranges as high as 0.5–1.5 g in multiple daily doses (Battu et al. 2010). Therefore, it is urgent necessary to find alternative formulation of berberine utilizing advanced drug delivery system. Liquid crystalline nanoparticles (LCNs) offer versatility in designing and delivery of drug targeting chronic respiratory disease (Chan et al. 2021). We have previously shown that Rutin and Naringenin-loaded LCNs offer better activity against oxidative stress and inflammation than using power form of these compounds (Mehta et al. 2021b; Paudel et al. 2020; Wadhwa et al. 2021). In line with the same hypothesis, we have designed berberine-loaded LCNs and explored the activity against lung cancer proliferation and migration. To improve the physiochemical properties and enhanced efficacy, our berberine LCNs formulation were tested for various characterization parameters (Tables 1 and 3, Figs. 5 and 6). These pharmaceutical tests showed a very favourable characterization data in terms of particle size, polydispersity index, entrapment efficiency, and in vitro release.

The molecular modelling simulations revealed two important findings. Firstly, the drug molecule was incorporated within the geometrical space of the lipid molecule as a strained bimolecular complex. Secondly, both the bi- and tri-molecular complexes were stabilized by van der Waals forces against all other bonding interactions (bond, angle, and dihedral energies). This is interesting because vdW energy component represents intermolecular interactions, which are non-covalent and reversible in nature. This is evident from the preferable positioning of the drug within the lipid molecule and further encapsulation into the MOO-PF127 system under formulation conditions. It must be noted that this vdW attraction also brought into strained network structures, which may ultimately be responsible for the release of the drug once the environment changes such as in release medium or in vitro cell growth medium and conditions (Huo et al. 2021).

Next, we performed in vitro biological activity of our formulation against A549 cells. For cancer/tumour progression, two major events (a) proliferation/growth and (b) migration/invasion facilitate the entire tumorigenesis. Our colorimetric MTT assay that measure the cell viability and further cell count with trypan blue assay showed significant inhibition of A549 in a concentration dependent manner with an IC_50_ value of 10.10 µM (Fig. 7). An in vitro study by Qi Hw et al. (2014) observed the IC_50_ of berberine powder to inhibit A549 cell proliferation using MTT assay was 56.15 ± 3.14 µM (Qi et al. 2014). Similarly, Chen et al. used 40–120 µM of free berberine power (dissolved in DMSO) to show significant anticancer activity in A549 cells by inhibiting cell proliferation, colony formation, migration (wound healing assay), and protein expression of BCL-2 and Bax (Chen et al. 2019). This suggests that our berberine-LCNs formulation was > 5 time more potent than free berberine powder. Inhibition of A549 cell growth was also observed in our colony formation assay where berberine-LCNs treatment at a dose of 2.5 and 5 µM significantly inhibited the colony formation (Fig. 9). Likewise, in our anti-migratory assay performed by measuring the wound closure after 24 h of berberine-LCNs treatment and migration of A549 cells in trans-well chamber (Boyden’s chamber) assay revealed potent anti-migratory activity of berberine-LCNs (Fig. 8A–D).

For mechanistic approach, we further elucidate the protein expression related with cancer cell migration and proliferation. Epithelial-mesenchymal transition (EMT) is a phenomenon where epithelial cells change to mesenchymal stem cells phenotype due to loss of their cell polarity and cell adhesion property that makes them more invasive and metastasis (Mittal 2016). Various proteins are involved in EMT in NSCLC that facilitate cancer cell invasion/metastasis (Tsoukalas et al. 2017a). Among these proteins, the role of SNAIL (Yang et al. 2017b), p27 (Zhao et al. 2015), and Vimentin (Tsoukalas et al. 2017b) to promote EMT has been widely studied. Silencing of SNAIL was correlated with suppression of tumour cell invasion by reversing EMT in NSCLC (Yang et al. 2017b) while there was negative correlation between vimentin expression and overall survival in NSCLC (Tsoukalas et al. 2017b).

**Fig. 12:**
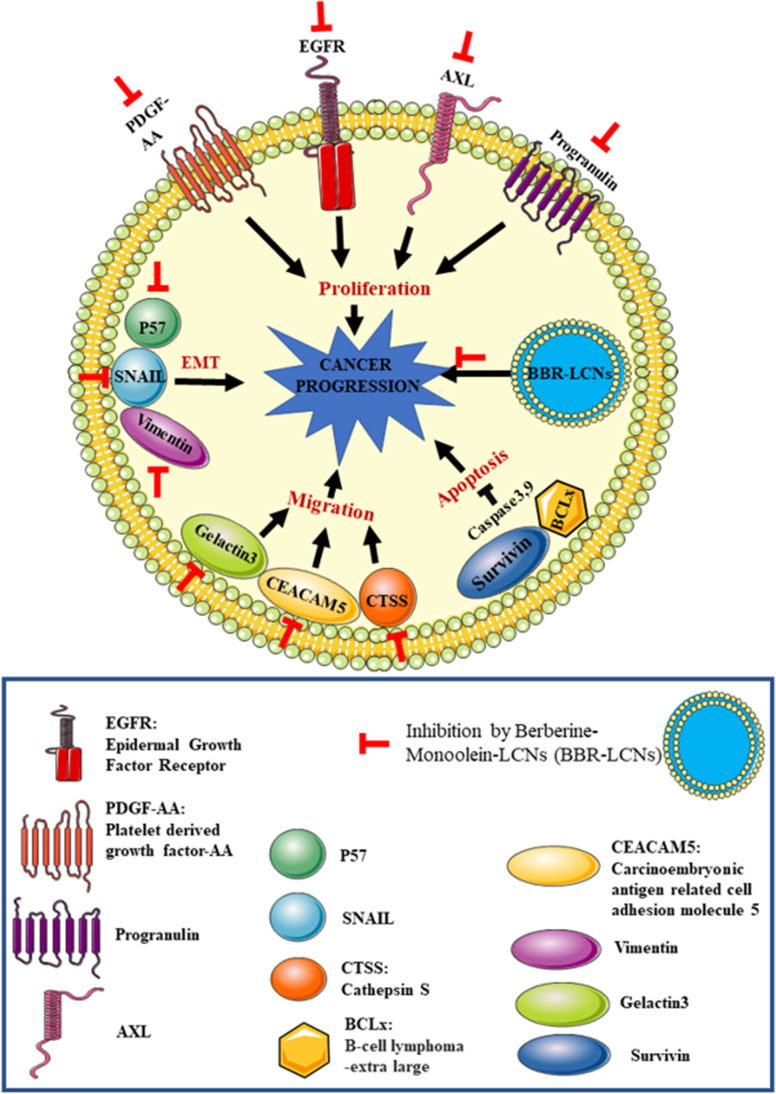
Anti-cancer mechanism of action of berberine LCNs. Lung cancer progression is a result of uncontrolled cell proliferation and migration. Proliferation of A549 cell is mediated overexpression of growth factor such as PDGF-AA and EGFR as well as Axl and progranulin protein. Similarly, migration of A549 is induced by upregulation of Galectin-3, CTSS, and CEACAM5 protein. Furthermore, metastasis of A549 cell is promoted by protein involved in EMT such as SNAIL, p27, and Vimentin. Protein such as survivin and BCLx increase cancer cell survival by resisting cell to undergo apoptosis. Berberine-LCNs showed anti-proliferative and anti-migratory activity by inhibiting aforementioned proliferation and migration-related protein respectively. Additionally, berberine-LCNs also inhibited protein involved EMT and protein make cancer cell resistance to apoptosis. Overall, the potent anti-cancer activity results in inhibition of cancer progression

In our study, treatment of berberine-LCNs at a dose of 5 µM significantly downregulated the protein expression of SNAIL, p27, and vimentin (Fig. 10a, b, and c). Likewise, certain proteins play key role in angiogenesis, proliferation, and survival of cancer cell (Fig. 12). Platelet-derived growth factor-AA (PDGF-AA) is crucial autocrine regulator of vascular endothelial growth factor expression in NSCLC, and it facilitates the process of angiogenesis (Shikada et al. 2005). Galectin-3 is another protein that promotes angiogenesis (Newlaczyl &Yu 2011) and metastasis (Reticker-Flynn et al. 2012). Similarly, Axl (an oncogenic protein) expression is observed in 60% of NSCLC cell line and it promote in cell proliferation as well as adhesion (Kim et al. 2015; Wimmel et al. 2001). Progranulin expression is correlated with epithelial cell growth including A549 and promotes tumour growth in vivo (He &Bateman 1999). ERBB3 belongs to the member of epidermal growth factor receptor (EGFR) family, and it plays a vital role in mediating NSCLC proliferation and differentiation (Guo et al. 2016). Protein such as Bcl-xL and Survivin are involved in cancer cell survival by inhibiting apoptosis of cancer cell (Hirano et al. 2015; Schott et al. 1995). Capthesin S (CTSS) protein is also strongly associated with NSCLC pathology owing to the fact that CTSS can degrade proteoglycan of interstitial matrix such as decorin (Kehlet et al. 2017) and nidogen-1 (Willumsen et al. 2017) to promote the NSCLC migration. Some protein such as CEA-related cell adhesion molecule 5 (CECAM5) stimulates both proliferation and migration of NSCLC (Zhang et al. 2020). In our protein array (Fig. 11), berberine LCNs treatment at 5 µM significantly inhibited the protein expression of PDGF-AA, Galectin 3, Axl, Progranulin, ERBB3, BCLx, Cathepsin S, Survivin, and CEACAM5. The details anti-cancer mechanism of action of berberine LCNs is shown in Fig. 12. Taken together, the anti-cancer activity of berberine LCNs against A549 cell was due to its potency to inhibited key protein involved in EMT, angiogenesis, metastasis, and proliferation.

There are few limitations as well as possibility of future direction from our study. Firstly, we can further prove the anti-cancer activity of berberine-LCNs through gene approach. In the current study, we have performed only protein-related work and therefore to investigate if berberine-LCNs inhibits key genes involved in A549 cell proliferation and migration would be interesting. Secondly, our study is entirely in vitro, and this opens a new platform for further in-depth studies in in vivo pre-clinical animal models of lung cancer. Third, we can also test the anti-cancer activity of berberine-LCNs in other cell line of lung cancer such as Calu3 and H460. Nevertheless, berberine can a potential therapeutic alterative for lung cancer if we can formulate it appropriately using advance drug delivery system and manipulate the physiochemical parameters to increase efficacy, stability, and cellular uptake.

## Conclusion

The advantage of formulating berberine powder into LCNs is clearly observed through potent anti-cancer activity in vitro against A549 cell line. The promising anti-proliferative and anti-migratory activity of berberine-LCNs was most likely due to significant inhibition protein involved in the EMT pathway that facilitate metastasis and other proteins involved in cell signalling pathway that leads to uncontrolled proliferation of cancer cells. The physiochemical parameter and molecular modelling also support the excellent design of drug delivery to increase cellular uptake and drug release. Further in-depth mechanistic insights on how berberine-LCNs inhibit cancer progression are necessary to scientifically validate berberine as a promising alternative approach for lung cancer. Our study opens platform for researchers to utilize advance cellular and molecular techniques to delineate the anticancer possibility of berberine through different approach such as silencing or knockout of gene and in-vivo studies to determine the treatment safety and efficacy. Our study could be interesting to nanobiotechnology-based biomedical/pharmaceuticals industry to test similar nanoformulation of compounds with poor solubility and bioavaibility for various forms of cancer.

## Data Availability

Not applicable.
